# Alignment of Community Pharmacy Foundation Grant Funding and the Evolution of Pharmacy Practice in the United States of America

**DOI:** 10.3390/pharmacy7020063

**Published:** 2019-06-14

**Authors:** Brittany Hoffmann-Eubanks, Anne Marie Kondic, Brian J. Isetts

**Affiliations:** 1 Banner Medical LLC, Frankfort, IL 60423, USA; 2 Community Pharmacy Foundation, Chicago, IL 60680, USA; amkondic@CommunityPharmacyFoundation.org; 3 College of Pharmacy, University of Minnesota, 308 Harvard St., SE, Room WDH 7-125-c, Minneapolis, MN 55455, USA; isett001@umn.edu

**Keywords:** community pharmacy, pharmaceutical care, medication therapy management, pharmacy practice, pharmacy education, grants

## Abstract

The Community Pharmacy Foundation is a non-profit organization dedicated to the advancement of community pharmacy practice and patient care delivery through grant funding and resource sharing. Since 2002, CPF has awarded 191 grants and over $9,200,000 (US dollars) in research and project grants. The purpose of this manuscript is to highlight the evolution of pharmacy practice and pharmacy education in the United States through the presentation of exemplary cases of Community Pharmacy Foundation funding that is aligned with new care delivery models and approaches to the advancement of patient-centered pharmacy care. Pharmacy began in colonial America as the United States of America was just beginning to form with apothecary shops and druggists. Over time, the pharmacy industry would be revolutionized as America became urbanized, and drug products became commercially produced. The role of the pharmacist and their education evolved as direct patient care became a clear expectation of the general public. By the 1990s, the pharmacy profession had carved out a new path that focused on pharmacist-led, patient-centered pharmaceutical care and medication therapy management services. The Community Pharmacy Foundation grant funding has aligned with this evolution since its founding in 2000, and multiple exemplary grants are presented as support. As the role of pharmacists again transitions from a fee-for-service model to a value-based model, the Community Pharmacy Foundation continues to provide grant funding for research and projects that support the advancement of community pharmacy practice, education, and expanded training of pharmacists.

## 1. Introduction

The Community Pharmacy Foundation (CPF) is a non-profit organization founded in 2000 that is dedicated to the advancement of community pharmacy practice and patient care delivery through grant funding and resource sharing [1]. CPF was originally founded as part of a federal settlement on behalf of community pharmacies in the United States (US) from class action litigation against discriminatory drug pricing. As a result, CPF was originally governed by a court-appointed Board of Directors (BOD) that consisted of four community pharmacists and a retired judge of the United States District Court in Illinois. As part of the settlement, $18.6 million (US dollars) were awarded to establish the CPF to advance the profession of community pharmacy. In the first two years after CPF was founded, the BOD established a formal process for the receipt, review, evaluation, awarding, and monitoring of grant dollars based upon submission of grant application requests [1].

The primary goals of CPF grant funding are:

1. The development, processing, and use of findings that affirm the value of community pharmacy practice in the healthcare delivery system.
2. The measurement, publication, and dissemination of findings documenting the value of professional services delivered to patients by community pharmacists.
3. Efforts that measure the impact of pharmacist interventions in achieving the targeted therapeutic goals set collaboratively by the patient, the pharmacist, and other members of the healthcare team.
4. Efforts that evaluate patient-specific outcomes with regard to the quality of care delivered by community pharmacists.

In January of 2002, CPF received its first grant submissions, and awarded its first two grants in May, 2002. Since 2002, CPF has awarded 191 grants and over $9,200,000 (US dollars) in research and project grants for the advancement of community pharmacy practice and patient care delivery improvement. The extent to which CPF grants have aligned with the primary goals of CPF has been reviewed in detail elsewhere [2,3]. In general, CPF grants in the early years (2002–2008) primarily focused on adding to the knowledge base of medical conditions and medications only, but have since (2009–2019) moved toward studies that affect health outcomes and change pharmacy practices [2]. This evolution of grant funding is in line with changes in the scope of practice of American pharmacy and pharmacists since its founding.

The purpose of this manuscript is to highlight the evolution of pharmacy practice and pharmacy education in the United States through the presentation of exemplary cases of the Community Pharmacy Foundation funding that is aligned with new care delivery models and approaches to the advancement of patient-centered pharmacy care.

## 2. Colonial America: The Roots of Pharmacy Practice in the United States

In Colonial America, there were very few standalone pharmacies. The first documented “pharmacist” was Bartholomew Browne of Salem in 1698 [4,5]. He operated a daily pharmacy and traded produce and merchandise in exchange for services. Still, Mr. Browne’s pharmacy was not the norm, as most physicians had their own apothecary shops and only utilized the druggist to purchase the raw materials required for compounding medicines [4,5]. Similarly, the majority of drugs utilized for compounding were imported from England. It was not until the Revolutionary war that apothecaries became more common, due to a need for domestic sources of medicine [6]. The products that apothecaries stocked also became more standardized, and typically included drugs, medicines, surgical supplies, dyestuffs, essences, and chemicals [4].

Combined physician medical practice and apothecary shops had been widely debated in England and were considered a conflict of interest [5]. However, the idea of separating physician and apothecary practices in colonial American was not introduced until the late 18th century by John Morgan [5]. He proposed the idea after traveling to Europe where drug distribution and physician practices had been separated to avoid conflicts of interest [5]. This concept was not popular and was widely rejected, since drug distribution was a large part of physicians’ livelihoods, and patients did not want the extra expense of seeing two practitioners [5]. It was not until the 19th century (after the war of 1812) that the preparation of medicine became the pharmacist’s responsibility and physician drug shops were phased out; however, very few physicians actually gave up their dispensing practice [5].

Pharmacy education was also evolving as American apothecary shops began to pop up across the country. For example, the first diploma for apothecary practices was awarded in 1808 by the Legislature of the territory of Orleans, and in 1818, South Carolina was the first state to require a pharmaceutical examination before obtaining a pharmaceutical license [4]. In December of 1820, the first official print of the Pharmacopoeia of the United States of America was published in English and Latin [4]. Also, state-affiliated pharmacy schools were created, with the Philadelphia College of Pharmacy (1821) and the Massachusetts College of Pharmacy (1823) being the first two in America [6]. Furthermore, these state-affiliated colleges had legislative curriculum requirements to ensure quality [6].

In the 1840s, inferior drug products were being shipped from Europe that led to the Drug Importation Act of 1848 [6]. This act led to drug examiners being stationed at points of entry to verify the quality, purity, and fitness of drug products being imported to America. Due to political cronyism, incompetent drug inspectors led to the failure of the Drug Importation Act and calls for a national pharmacy organization began to grow [6]. As a result, a group of 21 pharmacists and non-credentialed apothecaries met to form the American Pharmaceutical Association now called the American Pharmacists Association (APhA) on 6 October 1852 [7]. By the 1880s, the practice of pharmacy was recognized as an important public service, serving as the foundation for modern pharmacy practice in the United States.

## 3. CPF Grant Funding Aligns with the Evolution of Pharmacy Practice in the United States

**Figure 1:**
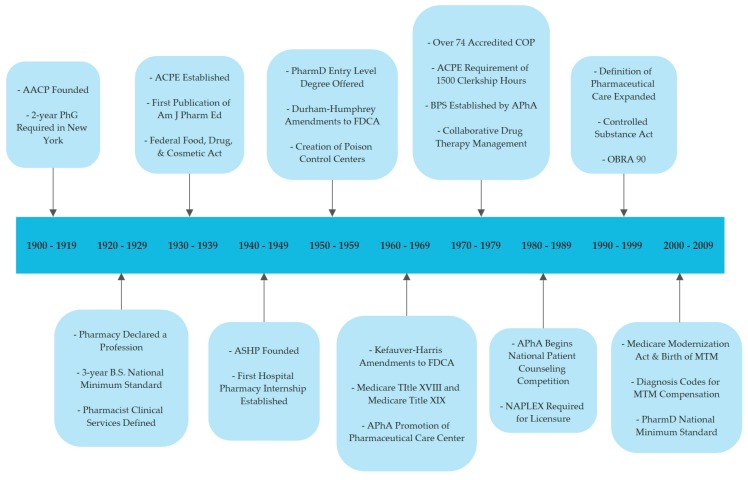
The Evolution of Modern Pharmacy Practice in the United States [4]. AACP: American Association of Colleges of Pharmacy; PhG: Pharmacy Graduate; B.S.: Bachelor of Science; COP: Colleges of Pharmacy; ACPE: American College of Pharmacy Education; Am J Pharm Ed: American Journal of Pharmacy Education; ASHP: American Society of Health System Pharmacists; PharmD: Doctor of Pharmacy; FDCA: Federal Food, Drug & Cosmetic Act; APhA: American Pharmacists Association; BPS: Board of Pharmacy Specialties; NAPLEX: National Association of Pharmacy Licensure Exam; OBRA: Omnibus Budget Reconciliation Act; MTM: Medicare Therapy Management.

By the end of the 19th century, the practice of pharmacy was significantly changing. Parke, Davis, & Company created the first standardized pharmaceutical extract (Liquor Erogtae Purificatus) in 1879 that revolutionized the business of pharmacy [8]. Instead of pharmacists compounding medications, a growing list of chemical companies began quickly expanding their research and development programs to develop and commercialize standardized drug products and delivery systems. For example, gelatin capsules became commercially available in 1875 followed by tablets and enteric coated tablets in 1884 [8]. Therefore, the role of the pharmacist shifted from primarily being a medication preparer to dispenser of commercially prepared medication. The 20th century would again bring further changes as a result of several factors, including increased urbanization in America, expansion of the manufacturing of drug products, and the emergence of new pharmacy business models (Figure 1).

As these changes were occurring, so too was the education of pharmacists within the United States. In the 1950s, the entry-level degree for pharmacists transitioned to a professional degree, which has been the standard since 2000 [4]. Furthermore, as the scope of practice for pharmacists began to evolve, so did the training required to meet those demands when pharmacists graduated and entered into practice. As such, the development of post-graduate hospital training programs was created in the early 1960s by the American Society of Health System Pharmacists (ASHP) [9]. Then in 1973, APhA established the Board of Pharmacy Specialties (BPS) which helped pave the way for post-graduate residency training in specialized practice areas [9]. For the next 20 years, hospital-based residencies with specific accreditation standards were implemented in health systems across the United States. By the 1980s calls began to grow for the creation of community pharmacy residency programs to improve clinical skills and prepare pharmacists for pharmacy ownership. In 1986, APhA created the pharmacy residency program initiative starting the process for developing community pharmacy post-graduate training programs [10]. By 1988, 200 ASHP residency programs had been accredited with 124 being in hospital pharmacy, 39 in clinical pharmacy, and 37 in specialty areas such as psychiatry and hospital administration. Furthermore, over 5800 residents had successfully graduated from an accredited ASHP residency program [9].

### 3.1. The Pharmaceutical Care Era

The term “pharmaceutical care” was first used in 1975, referring to the care a given patient requires and receives which assures safe and rational drug use [11,12]. The term manifested in response to renewed interest in shifting back to care for patients as opposed to the drug product, and coincided with the establishment of the clinical pharmacy era which recognized the value of pharmacists for their expert drug therapy knowledge [11]. The path of clinical pharmacy would be further developed in 1990 after a landmark article by Hepler and Strand was published outlining the patient care responsibilities of pharmacists that went beyond the traditional role of dispensing [13]. As a result, a new professional practice emerged that required a definition to aid in moving the idea from theory into application [11]. Thus, a pharmaceutical care practice is one in which the practitioner takes responsibility for all of a patient’s drug-related needs and is also accountable for this commitment [11,13]. However, at the time, there was not an overall consensus for the best way to accomplish this expanded role of the pharmacist.

Therefore, initial demonstration projects and pilot programs were conducted to help add to the knowledge base of pharmacists as healthcare providers. For example, CPF awarded its very first grant in May of 2002 for a program that sought to evaluate the effectiveness of a community pharmacist-based home blood pressure monitoring program [14]. The study included 125 patients from 12 different community pharmacies. Patients were randomized to receive a high-intensity or low-intensity blood pressure intervention to determine differences in systolic and diastolic blood pressure from baseline to follow-up between the high-intensity and low-intensity patients. The results showed the high-intensity intervention achieved a lower diastolic blood pressure (*p* = 0.03) and could serve as a strategy for future patients with hypertension [14].

Another early study funded by CPF in 2002 examined pharmacy participation in the Wisconsin Medicaid Pharmaceutical Care Program (WMPCP) [15]. Pharmacies who participated in this program were provided an enhanced dispensing fee for pharmaceutical care actions. This study retrospectively reviewed administrative claims paid between 1997–2003 to investigate characteristics of claims submitted to WMPCP and claims paid for pharmaceutical care services. There were 359 pharmacies who participated in WMPCP for seven years. The claims per pharmacy ranged from 9.41–64.37, with an increasing volume trend observed at the end of the study period. The results showed that patient behavior, pharmacists resolving problems with patients, and patient response were the most common claims. Furthermore, some of the pharmacies had incorporated the program into their practice routinely. Also, pharmacists focused on drug therapy problems related to patient behavior, and worked directly with the patient to resolve these problems through education [15].

As more evidence became available about the value of pharmacists in helping improve patient outcomes expanded care delivery models began to be created. For example, CPF funded a study grant that was completed in 2006 and evaluated the effects of an extended diabetes care program compared to regular care on primary clinical outcomes such as hemoglobin A1c, low-density lipoprotein (LDL) cholesterol, and blood pressure [16]. The extended care diabetes program was provided by trained community pharmacists over 12-months with quarterly visits. The extended care program consisted of drug-therapy evaluation, patient education, and drug-therapy recommendations to the patient’s physician as needed. Twelve pharmacists and eight community pharmacies participated in the program. A total of 67 subjects completed the study. The results showed pharmacist intervention helped to decrease LDL cholesterol levels significantly and resulted in a significant improvement in aggregate patient self-management of diabetes. There was no significant impact observed for blood glucose control or blood pressure levels. However, the overall program results were positive and helped to decrease the cardiovascular risks of the enrolled patients [16]. Additional CPF grants were awarded during the pharmaceutical care era that focused on pharmacy operations and furthering our understanding of the pharmacists’ impact on disease state management [17,18,19,20,21].

At the same time, pharmacy education was also evolving to keep up with the changes in pharmacy practice. In 1997, APhA updated their post-graduate year-one (PGY1) community pharmacy residency program guidelines to reflect the new focus on pharmaceutical patient care [10]. These revised guidelines were then used to develop the first APhA-ASHP community pharmacy residency accreditation standards that were adopted in 1999. The adoption of the accreditation standards was an important step in the evolution of community pharmacy and for the development of community pharmacy residency programs. Further, accreditation standards increased program development, increased the perceived value of community pharmacy residencies, and allowed eligible student pharmacists to participate in the Residency Match Program [10].

### 3.2. Relationship of Medication Therapy Management to the Practice of Pharmaceutical Care

Medication therapy management (MTM) was the next development in the evolution of pharmacy practice. Origins of the term Medication Therapy Management can be traced to legislation proposed in the mid-1990s intended to reimburse pharmacists for pharmaceutical care services [22]. Then in 2002, the Medicare Payment Advisory Commission (MedPAC) prepared a report for Congress on Medicare payment for nonphysician providers describing medication management as an evolving approach in which drug therapy decisions are coordinated collaboratively by physicians, pharmacists, and other care providers together with the patient [23]. Shortly after this MedPAC Report was published, there were several noteworthy events that clarified MTM as a service provided within the practice of pharmaceutical care. A coalition of national pharmacy organizations collaborated with the American Medical Association’s Current Procedural Terminology (CPT^®^) Editorial Panel in preparation of a proposal establishing reporting and billing codes for pharmacists’ clinical services. In official health reporting nomenclature of CPT^®^, Medication Therapy Management Services (MTMS) is described as face-to-face assessment and intervention as appropriate, by a pharmacist, to optimize the response to medications or to manage treatment-related medication interactions or complications [24]. Furthermore, the comprehensiveness of MTMS in official health reporting nomenclature was further clarified as an assessment to identify, resolve and prevent drug therapy problems, formulating a medication treatment plan to achieve patients’ goals of therapy, and follow-up monitoring and evaluation of patient outcomes [24,25]. CPF also supported a grant to develop educational tools to advance MTMS [26].

MTM service has also been characterized by the Centers for Disease Control and Prevention (CDC) as “a distinct service or group of services provided by healthcare providers, including pharmacists, to ensure the best therapeutic outcomes for patients. MTM includes five core elements: medication therapy review, a personal medication record, a medication-related action plan, intervention or referral, and documentation and follow-up” [27]. In addition, this service was also recognized in the Medicare Modernization Act of 2003 (MMA), which established the Medicare Part D drug benefit and requirements for controlling costs, quality improvement, and MTM programs [28]. As part of the MMA, broad goals were established to optimize therapeutic outcomes (e.g., reducing adverse events) for targeted beneficiaries. Afterward, several pilot programs were carried out across the US to increase knowledge of MTM (e.g., Asheville Project, Diabetes Ten City Challenge) [28]. These early pilot programs were essential in the process of creating a profession-wide-consensus about the process of MTM [29].

CPF’s grant funding during this time also went through an evolution process by moving towards the funding of projects that were more outcomes based. A study by Isetts et al. compared the impact of CPF funding between the initial years and recent years of the foundation utilizing the AHRQ Impact Factor Framework (Table 1). Their study found a trend of more recently completed grants having a higher AHRQ impact level compared to those in the initial years. For example, 53% of projects completed in the initial years had an AHRQ impact of level 1 compared to 36% in recent years. Furthermore, grants completed more recently were more likely to have a higher impact at levels 2, 3, and 4 (36%, 26%, 3%, respectively) [2].

CPF grants during the early years of MTM also helped to contribute to a professional consensus about the viability of MTM. For example, CPF funded a grant that was completed in 2008 that evaluated the effect of implementing MTM services (pharmaceutical case management [PCM]) conducted by community pharmacists for a private health plan [30]. The PCM model had already demonstrated success with Iowa Medicaid beneficiaries and was an innovative model of care when initiated in 2000. Therefore, an 18-month pilot study was conducted with CPF support to assess the benefits of implementing the PCM model in a private sector health plan. The same PCM model used for Iowa Medicaid Beneficiaries was adopted; however, only pharmacists could be reimbursed in this program (including collaboration with some case managers and disease managers) [30].

The objectives of the PCM pilot program were to evaluate the effect of PCM on medication appropriateness, characterize drug-therapy problems identified by PCM pharmacists, compare healthcare utilization among patients eligible for PCM services, assess the impact of PCM on patient’s self-reported health, and assess pharmacist barriers to providing PCM services [30]. The pilot program showed on average almost three drug-therapy problems were identified per patient over the study period, and 89.3% were resolved by the pharmacist. This early pilot program identified significant barriers such as the higher health status of private sector patients which may have led to a perception that they did not need the PCM service, and 50% of pharmacies had less than five eligible patients. Overall, PCM services were considered an opportunity to reduce overall healthcare costs and utilization; and served as a launching pad for future MTM studies conducted in the community pharmacy setting [30].

Another CPF grant awardee showed it was possible to set up a network of 135 pharmacists across seven states of the upper Midwest to provide MTM services to older adults [31]. As a result, the grantee was able to create a tool, called TIMER (Tool to Improve Medications in the Elderly via Review), which was shown to be helpful for pharmacists to provide MTM services to older adults [31]. There are multiple other examples of CPF funded grants in the early MTM era which also contributed to the knowledge base of pharmacists providing MTM services in the community pharmacy setting [32,33,34,35,36].

The funding of expanded MTM services with greater disease state complexity became commonplace after the viability of pharmacist-led MTM programs was established based upon CPF grant funding. For example, a grant completed in 2009 implemented a pharmacist-led rapid strep test service in the community pharmacy setting [37]. Furthermore, the participating pharmacists also obtained prescriptive authority via collaborative practice agreements in order to provide antibiotic treatment after a positive rapid strep test. In total, 85 rapid strep tests were performed and showed pharmacists could conduct rapid strep tests, identify the presence or absence of group A Streptococcus, and whether or not antibacterial therapy should be initiated [37].

Similarly, a 2014 pilot project evaluated whether or not community pharmacists could identify patients at risk of worsening heart failure through the use of a clinical decision tool called the One Minute Clinic for Heart Failure (TOM-C HF) [38]. TOM-C HF was a simple six-item screening tool that would be used during routine patient encounters. Results showed application of the screening tool took about 1 to 5 min in over 80% of the patient interactions. Furthermore, of the 121 patients evaluated, 62% had one or more symptoms of worsening heart failure. The most common symptoms identified were edema (39%) and increased shortness of breath (17%). Also, self-reported weight gain (>5 lbs) was also found in 19% of patients. This study showed that pharmacists can help screen and identify patients for heart failure decompensation and may be an important link in disease state management of heart failure to help lower hospital readmission rates [38].

The establishment of community pharmacy residency programs also began to grow rapidly during this time, with the number of programs almost tripling between 1999 and 2001 [10]. This growth has been attributed to an increase in clinical services being offered in the community setting, increased buy-in from colleges of pharmacy, and significant funding for PGY1 residency programs by the Institute for the Advancement of Community Pharmacy (IACP). IACP provided over $900,000 (US dollars) and led to the establishment of 45 community pharmacy residency positions over six years [10]. CPF grant funding was also in line with the trend of increased focus on the value of community pharmacy residencies during the MTM era and in 2004, CPF partnered with the APhA Foundation to co-fund community pharmacy resident incentive grants to cultivate innovative research projects of pharmacy residents (https://www.aphafoundation.org/incentive-grants) [39].

In 2010, CPF funded a grant that surveyed the perceived value of providing community pharmacy residency training from the perspective of the colleges of pharmacy and pharmacy provider organizations [40]. Survey findings revealed the most common value responses were altruistic (e.g., pharmacy profession development and pharmacy education development). In addition, barriers to offering residency programs included operational issues and challenges related to accreditation [39]. In 2014, CPF also funded a project that created an implementation guide for PGY1 community pharmacy residency programs to support the growth and expansion of these programs and to contribute to the advancement of community pharmacy [41]. As a result of this grant, the “implementation guide provided critical resources and materials to assist organizational entities, community pharmacies, and colleges of pharmacy in their development, implementation, and accreditation of new community pharmacy residency programs” [41].

### 3.3. The Value-Based Care Era

Pharmacists are considered the most accessible healthcare provider, and are uniquely qualified to provide patient-centered care that engages patients in proper medication use and chronic disease state management [42]. The provision of MTM services within the practice of pharmaceutical care forever changed the way pharmacists would be involved in the care of patients with expanded roles such as patient assessment, identification of therapeutic interventions, medication synchronization, immunizations, point-of-care testing, chronic disease state management, and therapeutic monitoring and follow-up. As such, the trend in the healthcare industry has seen a movement from a fee-for-service care model to one that is value-based where patient outcomes result in pay-for-performance as opposed to the volume of services rendered [42]. Pharmacists have begun to take on even more responsibility in the care of patients concerning medication optimization, clinical status, patient satisfaction, and chronic disease state outcomes. As a result, there are expanded opportunities for pharmacists to partner with physicians, payers, and other healthcare stakeholders more than ever before to improve health outcomes, enhance the patient experience, and reduce the total cost of care.

Approximately 60% of the grants awarded by CPF are direct-patient care focused. For example, a CPF grant completed in 2017 evaluated the effects of individualized patient care services by pharmacists on total costs of care, an adherence measure, and the use of high-risk medications by older patients [43]. To accomplish this, the grantee setup a separate pharmacy-based medical clinic that was staffed by healthcare providers who were recognized by payers to bill for services. The pharmacists, in collaboration with the rendering providers, were able to increase the number of patients seen, the quality of patient clinical outcomes, and the number of clinical services the pharmacy offers. The clinic saw 309 patients between September 2014–December 2015. An A1c drop of −1.29 was observed (average 8.72 to 7.43), and the patient’s body mass index dropped −3.28 (35.68–32.4) after six months of the intervention. As a result of the demonstrated value for services, the clinic pharmacy was able to establish a limited provider status with a large healthcare payer leading to direct pharmacist billing and payment (vs. incident-to-billing) [43].

Another CPF grant completed in 2017 utilized targeted medication reviews by pharmacists to deliver pre-conception care as a public health demonstration project [44]. Community pharmacists were provided an American College of Pharmacy Education (ACPE) accredited continuing education program on addressing pre-conception care needs of patients via the framework of MTM. The targeted medication reviews (TMRs) included women 15 to 45 years enrolled in an Ohio managed care plan and focused on medications that could cause fetal harm, folic acid use, and immunizations. TMRs were generated and assigned to pharmacies where the eligible patients filled prescriptions. Any pharmacy in Ohio that participated in the commercially available MTM platform received a TMR notification. Pharmacists then provided the service, documented, and billed for the service through the MTM platform. Results showed 1149 pharmacists from 818 pharmacies completed at least 1 TMR (n = 6602). The TMRs had a 33% completion rate with a 65% (n = 4266) success rate. This demonstration project showed new service programs could be rapidly implemented into existing MTM service processes over hundreds of pharmacies in Ohio. Furthermore, it also provides data demonstrating the value of pharmacists and can serve as justification for additional payers to reimburse for similar services. Finally, pharmacists may be able to assist with other similar preventative care services and utilize this model to improve patient health outcomes as well as obtain reimbursement for their service [44].

Value-based services will require the collaboration of multiple healthcare providers including pharmacists to help patients achieve the best health outcomes. To better understand this requirement, a CPF grant completed in 2017 was conducted to increase knowledge about collaborative working relationships between pharmacists and other healthcare providers throughout multiple practice settings [45]. The study sample included 16 community pharmacists, nine prescribers, and five care managers. There was an agreement between all participants for the need to have face-to-face meetings with other healthcare team members to determine shared goals. Prescribers and care managers identified that community pharmacists providing education on medications was very helpful and also helped them reinforce the education each time they saw a patient. Also, medication adherence packaging provided by community pharmacists was also stated as helpful in improving adherence to chronic medications per prescribers and case managers. Community pharmacists from the project felt personally responsible for a patient’s medication regimen. Also, prescribers and care managers felt they had additional time to focus on other, non-medication related issues when they worked with a community pharmacist to manage the patient’s medication regimen. As a result, this demonstration project showed that including community pharmacists into team-based care can potentially improve patient care and outcomes [45].

CPF grants supporting pharmacists in patient-centered medical homes (PCMH) are important for the understanding of community pharmacist roles and responsibilities in alternate payment models [46,47,48,49]. The collaboration of pharmacists with other interprofessional care teams has further expanded during the value-based care era with the creation of alternative payment models in both commercial and government health insurance programs, such as accountable care organizations (ACOs). An ACO according to the Centers for Medicare and Medicaid Services (CMS) is a “group of doctors, hospitals, and other healthcare providers who come together voluntarily to deliver coordinated high-quality care to Medicare patients” [50]. This coordinated care helps reduce healthcare costs and ensure patients are receiving proper care while preventing unnecessary duplication of services and preventing medical errors. Further, when ACOs are successful in providing coordinated care for chronic disease states while simultaneously lowering costs they share in the savings they achieve for the Medicare program [50].

CPF grant funding has also been awarded to projects that seek to evaluate and establish the role of community pharmacists in an ACO. For example, a grant completed in 2016 assessed the feasibility of integrating MTM services provided by community pharmacists into the clinical care teams and the health information technology (HIT) infrastructure for a Minnesota Medicaid ACO [51]. The study included 15 community pharmacies who were all integrated into the HIT infrastructure via Direct Secure Messaging. There were 32 recipients who received MTM services resulting from ACO referral at 5 out of the 15 community pharmacies over one year. The project was able to set up an electronic MTM referral system successfully, and there was consideration given to the community pharmacists providing MTM in future ACO shared savings agreements [51].

Pharmacy education has also continued to evolve as pharmacists take on more advanced patient care roles. Since June of 2013, 34,824 pharmacists have graduated from ASHP accredited residency programs [9]. Also, BPS now recognizes twelve distinct specialties and more than 41,000 pharmacists, national and internationally, are board certified [52]. CPF continues to fund grants that support pharmacy education and the expansion of community pharmacy practice. For example, a 2016 CPF grant supported the development of a vision and strategic action plan for the future of community-based residency training [53]. This funding is directly aligned with the further evolution of pharmacy practice and the goals of achieving provider status and expanding access to care. The strategic action plan will help ensure the future needs of community-based pharmacist practitioners are met [53].

## 4. Conclusions

In summary, the Community Pharmacy Foundation is a non-profit organization whose mission is to support the advancement of community pharmacy practice and patient care delivery through grant funding and resource sharing. The history of pharmacy within the United States dates back to the beginning of the colonies, when physicians, druggists, and apothecaries were essential to providing health care in the new world. As America became urbanized and continued to grow in population, the necessity for domestic sources of drugs and pre-manufactured medications drastically changed the business of pharmacy, placing the pharmacist in a dispensing role. However, the pharmacist’s extensive drug therapy knowledge would create an environment to shift the focus back onto the patient and create new pharmacy care service models that have been the backbone of modern pharmacy practice in the United States. Since its founding, CPF has actively supported grants that are aligned with the evolution of pharmacy practice and CPFs mission. In the early years of the foundation, the majority of grants focused on building the pharmacy knowledge base, but have since shifted towards impacting the health outcomes of patients. In addition, the CPF grants awarded have also helped contribute to establishing reimbursement models for pharmacies and pharmacists who participate in advanced patient care and the advancement of the profession through education and training.

## Figures and Tables

**Table 1:** The Four AHRQ Impact Factor Levels [2].

AHRQ Level	AHRQ Level Description
1	Studies that add to the knowledge base only and do not represent direct change in policy or practice
2	Studies that may lead to a policy or program change as a direct result of the research
3	Studies that may cause a potential change in what clinicians or patients do, or result in a change in a care pattern
4	Studies that may change actual health outcomes (clinical, economic quality of life, and/or patient satisfaction), or profoundly change practice
